# Inhibitory Effects of Pinostilbene on Adipogenesis in 3T3-L1 Adipocytes: A Study of Possible Mechanisms

**DOI:** 10.3390/ijms222413446

**Published:** 2021-12-14

**Authors:** You Chul Chung, Chang-Gu Hyun

**Affiliations:** Jeju Inside Agency & Cosmetic Science Center, Department of Chemistry and Cosmetics, Jeju National University, Jeju 63243, Korea; jyc8385@hanmail.net

**Keywords:** pinostilbene, resveratrol, 3T3-L1 cells, adipogenesis, MAPK, AKT, AMPK, CREB

## Abstract

Resveratrol is a phytoalexin with multiple bioactive properties, including antioxidative, neuroprotective, cardioprotective, and anticancer effects. However, resveratrol exhibits structural instability in response to UV irradiation, alkaline pH, and oxygen exposure. Thus, resveratrol derivatives have attracted considerable research interest. In this study, we aimed to evaluate the anti-adipogenic effects of pinostilbene hydrate (PH), a methylated resveratrol derivative, in 3T3-L1 cells. We also evaluated the mechanisms underlying the effects of PH on adipogenesis in 3T3-L1 adipocytes. Oil Red O staining, lipid accumulation assay, and triglyceride (TG) content assay revealed that PH significantly inhibited lipid and TG accumulation without cytotoxicity. In addition, we determined that PH decreased the expression of adipogenesis-related transcription factors, such as PPARγ, C/EBPα, SREBP-1c, and FABP4, and the phosphorylation of MAPK and protein kinase B (AKT). Moreover, PH attenuated the expression of CREB and C/EBPβ, while increasing the phosphorylation of AMPK and ACC, and decreasing the expression of fatty acid synthase and FABP4. Based on these results, we suggest that PH suppresses adipogenesis in 3T3-L1 cells via the activation of the AMPK signaling pathway and the inhibition of the MAPK and AKT insulin-dependent signaling pathways.

## 1. Introduction

Obesity is caused by the accumulation of excess calories in the body when the energy gained via the consumption of high-calorie foods exceeds its utilization [1]. If this energy imbalance continues, most of the excess energy is converted to triglycerides via fatty acids and accumulates in adipocytes, resulting in weight gain [2,3]. Early stages of obesity are characterized by an increase in adipocyte size (hypertrophy), whereas the later stages are marked by an increase in the number of adipocytes (hyperplasia) [4,5]. Hypertrophy and hyperplasia result in an abnormal increase in the levels of hormones and cytokines secreted from adipose tissue, leading to pathological conditions such as type 2 diabetes, hypertension, hyperlipidemia, cardiovascular disease, and cancer [6,7,8].

Adipose tissue, commonly known as body fat, is composed of adipocytes, which are formed by the differentiation of preadipocytes through the process of adipogenesis and store excess energy in the form of triglycerides [9]. Furthermore, when there is an energy deficit in the body, adipose tissue releases triglycerides, which are stored in adipocytes in the form of glycerol and fatty acids, to supply energy [10]. In addition to this energy storage function, adipose tissue plays a role in various biological processes, such as the immune response and glucose and lipid metabolism, and serves as an endocrine organ by secreting various hormones such as adiponectin, leptin, and adipsin [11,12,13,14,15,16]. Therefore, adipose tissue is highly important for maintaining physiological homeostasis.

Adipogenesis is an important process in adipocyte formation and lipid accumulation, and the major transcription factors regulating this process are CCAAT/enhancer-binding protein alpha (C/EBPα), peroxisome proliferator-activated receptor gamma (PPARγ), sterol response element-binding protein-1c (SREBP-1c), fatty acid synthase (FAS), and fatty acid-binding protein 4 (FABP4) [17,18]. C/EBPα and PPARγ are essential for the differentiation of precursor cells into mature adipocytes, and PPARγ is capable of promoting adipogenesis in cells lacking C/EBP expression. SREBP is a supplementary regulator of adipogenesis and plays a role in regulating lipid metabolism and FAS expression. These transcription factors regulate the synthesis of fatty acids and triglycerides during adipogenesis, and FAS and FABP4 also affect the later stage of adipocyte differentiation and are responsible for adipocyte formation [19,20,21].

The mitogen-activated protein kinase (MAPK) and protein kinase B (AKT)-related insulin signaling pathways are known to activate adipogenesis in adipocytes. Phosphorylation of members of the AKT and MAPK family, extracellular signal-regulated kinase (ERK), and p38 is independently induced by insulin [22]. Phospho-AKT activates SREBP-1c and subsequently regulates PPARγ activation [23,24]. Moreover, AKT stimulates the phosphorylation of cAMP response element-binding protein (CREB). Phospho-CREB induces the activation of C/EBPβ to increase the transcriptional activity of C/EBPα and PPARγ and ultimately induces adipocyte differentiation [25,26,27]. Additionally, phosphorylation of ERK and p38 leads to the activation of C/EBPα and PPARγ [28,29].

Recent research has revealed that AMP-activated protein kinase (AMPK) controls energy recognition and homeostasis in the body, playing an important role in carbohydrate and fat metabolism. AMPK is activated to maintain energy homeostasis when intracellular energy levels decrease due to metabolic stress or exercise, and abnormal AMPK expression is strongly associated with metabolic and cardiovascular diseases, and cancer [30]. AMPK plays an important role in the regulation of adipogenic metabolism by mediating the synthesis and degradation of fatty acids. AMPK is a member of the serine/threonine kinase and forms a heterotrimeric complex with one catalytic α subunit and two regulatory β and γ subunits [31]. This kinase is activated by phosphorylation of threonine 172, which is located in the catalytic domain of the α subunit. Activation of AMPK inhibits the synthesis of malonyl-CoA from acetyl-CoA through acetyl-CoA carboxylase (ACC) inactivation by phosphorylation and ultimately suppresses lipogenesis by inhibiting the expression of SREBP-1c, FABP4, and FAS [32,33].

Resveratrol (3,5,4′-trihydroxy-trans-stilbene) is a polyphenolic compound and a phytoalexin found in peanuts or red grapes and has been widely studied for its bioactive properties, such as its anti-inflammatory, antioxidative, neuroprotective, cardioprotective, and anticancer effects [34,35,36,37,38,39]. In addition, it has been determined that resveratrol exerts its inhibitory effect on adipogenesis by decreasing lipid accumulation in and proliferation of various cell lines [40]. However, UV expression, extreme pH conditions, and oxygen exposure make the structure of resveratrol unstable, resulting in reduced bioavailability and bioactivity. Therefore, many recent studies have focused on the bioactivity of resveratrol derivatives containing a methoxy group instead of a hydroxyl group [41,42,43,44].

Among the methylated derivatives of resveratrol, pterostilbene is being actively studied. Resveratrol and pterostilbene have many similar bioactivities, such as antioxidative [45], anti-inflammatory [46], anti-obesity [47,48,49], anticancer [50,51], and neuroprotective activities [52,53,54,55]. However, comparative studies of these two compounds have shown that the biological activities and oral bioavailability of pterostilbene are normally superior to those of resveratrol [45,46,50,51]. In metabolic studies, it was reported that pinostilbene is the major metabolite of pterostilbene, which is produced by the gut microbiota demethylase in the mouse colon [56]. In addition, pinostilbene was found to be more stable than resveratrol under conditions simulating the colon environment, using human fecal microbiota [57]. Moreover, pterostilbene showed similar inhibitory effects on the growth of human colon cancer cells [58].

Pinostilbene (3,4′-dihydroxy-5-methoxystilbene) is a methylated resveratrol derivative, the bioactivity of which has been studied. Pinostilbene hydrate (PH) has exhibited strong neuroprotective activity in SH-SY5Y cells [44], anticancer effects in LNCaP cancer cells [59], anti-metastatic effects in human oral cancer cells [60], anti-melanogenic effects in B16F10 and human epidermal melanocytes [61], and anti-oxidative and tyrosinase inhibitory activities [62]. However, the inhibitory effects of PH on adipogenesis and the mechanisms underlying these effects have not yet been investigated. Therefore, in this study, we verified the anti-adipogenic effects of PH in 3T3-L1 adipocytes and examined possible molecular mechanisms.

## 2. Results

### 2.1. Toxicity of PH toward 3T3-L1 Adipocytes

**Figure 1:**
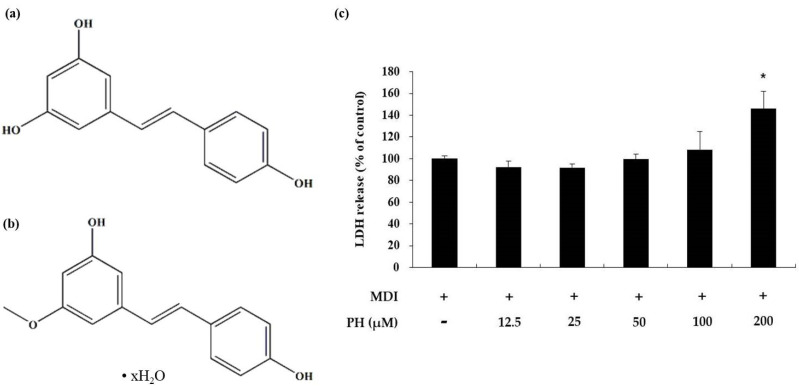
Effects of PH on cytotoxicity in 3T3-L1 adipocytes. The chemical structure of (**a**) resveratrol and (**b**) pinostilbene hydrate. Adipocyte differentiation was induced in post-confluent cells using IBMX/DEX/insulin (MDI) differentiation medium, and the cells were treated with the indicated concentrations of PH for 48 h. (**c**) Cytotoxicity was measured using the LDH assay. LDH release is expressed as percentages relative to the control group (MDI treated only). All values are presented as the mean ± standard deviation. (*n* = 3 per group). * indicates significantly different versus MDI alone (*p* < 0.05).

To determine the toxicity of PH toward 3T3-L1 adipocytes, a lactate dehydrogenase (LDH) assay was performed, where the LDH released into the medium was quantified 48 h after differentiation. There was no significant difference in the LDH content in the medium compared with that in the control cells following treatment with 12.5, 25, 50, and 100 μM PH. However, the LDH content in the medium increased by 46% compared with that in control cells following treatment with 200 μM PH (Figure 1c). Based on this result, PH was confirmed to be cytotoxic at 200 μM, and further experiments were performed using 12.5, 25, 50, and 100 μM PH.

### 2.2. Effect of PH on Lipid Accumulation and Triglyceride Content in 3T3-L1 Adipocytes

**Figure 2:**
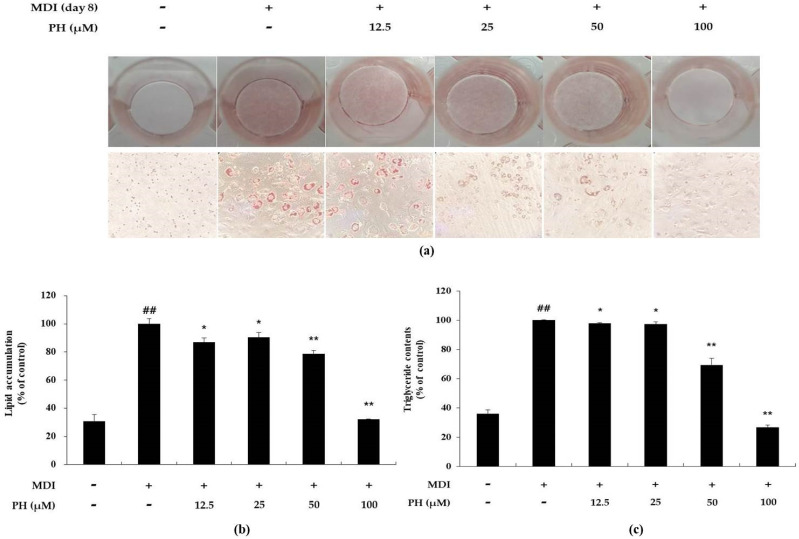
Effect of PH on lipid accumulation and triglyceride content in 3T3-L1 adipocytes. Adipocyte differentiation was induced in post-confluent cells using MDI differentiation medium, and the cells were treated with the indicated concentrations of PH for 8 days. (**a**) Differentiated adipocytes were stained using Oil Red O on day 8 and the cells were visualized by microscopy with 100× magnification. (**b**) The stained lipid droplets were dissolved using 2-propanol and measured using a spectrophotometer to quantify lipid accumulation. (**c**) The reduction in TG content was quantified using the EZ-triglyceride quantification assay kit. Lipid accumulation and TG content are expressed as percentages relative to the control group treated with MDI alone. All values are presented as the mean ± standard deviation. (*n* = 3 per group). * *p* < 0.05, ** *p* < 0.01 indicate significant differences versus the MDI-only control. ## *p* < 0.01 indicates significant differences versus an untreated control.

Lipid accumulation was measured and visually confirmed using the Oil Red O (ORO) staining assay. In the present study, to determine whether PH affects the inhibition of lipid accumulation, differentiation was induced in 3T3-L1 cells using a differentiation medium containing various concentrations of PH for 8 days, following which the ORO staining assay was performed. Lipid accumulation was markedly higher (3.2-fold) in differentiation-induced adipocytes than in untreated control cells. However, PH decreased lipid accumulation by 13.2%, 9.4%, 21.3%, and 67.9% at 12.5, 25, 50, and 100 μM, respectively, during adipocyte differentiation (Figure 2b). A triglyceride (TG) content test was performed to determine whether PH also reduced TG content in mature adipocytes. TG content was markedly higher in mature adipocytes, whereas it was reduced by 2.2%, 3.1%, 31.4%, and 72.6% in the presence of PH 12.5, 25, 50, and 100 μM, respectively, compared with that in mature adipocytes (Figure 2c).

### 2.3. Effect of PH on the Expression of Adipogenesis-Related Transcription Factors

**Figure 3:**
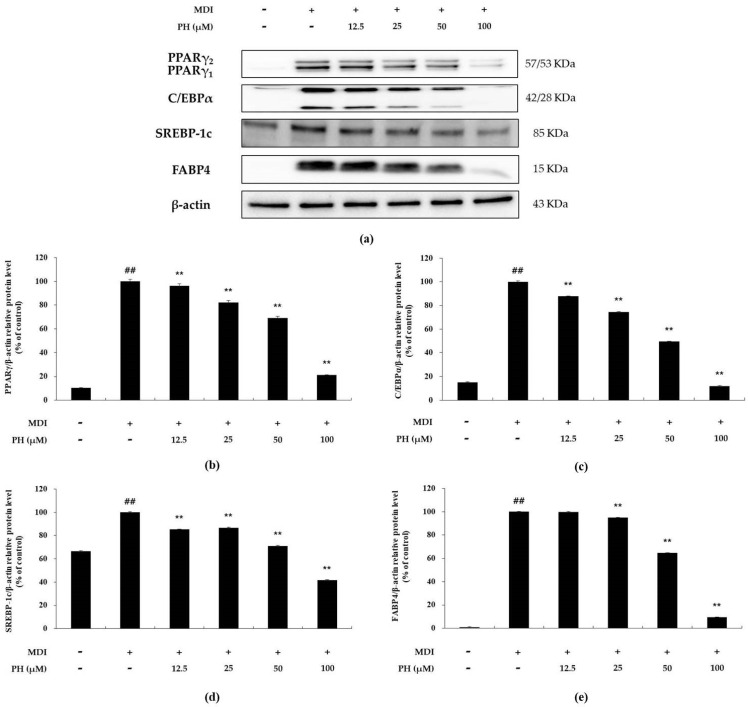
Effect of PH on the expression of adipogenesis-related transcription factors. Adipocyte differentiation was induced in post-confluent cells using MDI differentiation medium and treated with the indicated concentrations of PH for 6 days. (**a**) Western blot of PPARγ, C/EBPα, SREBP-1c, FABP4, and β-actin in 3T3-L1 adipocytes. The relative protein levels of (**b**) PPARγ/β-actin, (**c**) C/EBPα/β-actin, (**d**) SREBP-1c/β-actin, and (**e**) FABP4/β-actin are presented as the mean ± standard deviation of three independent measurements using the ImageJ program. The results in the relative protein levels are expressed as percentages relative to the control group treated with MDI alone. ** *p* < 0.01 indicates significant differences versus the MDI-only control. ## *p* < 0.01 indicates significant differences versus an untreated control.

To examine the effect of PH on the expression of adipogenesis-related transcription factors in differentiated 3T3-L1 adipocytes over 6 days, Western blot analysis was performed. The expression of these transcription factors, including PPARγ, C/EBPα, SREBP-1c, and FABP4, was higher in differentiating adipocytes than in untreated control cells. However, the protein levels of these adipogenic regulators were reduced after treatment with the indicated concentrations of PH (Figure 3).

### 2.4. Effect of PH on the Phosphorylation of MAPK

**Figure 4:**
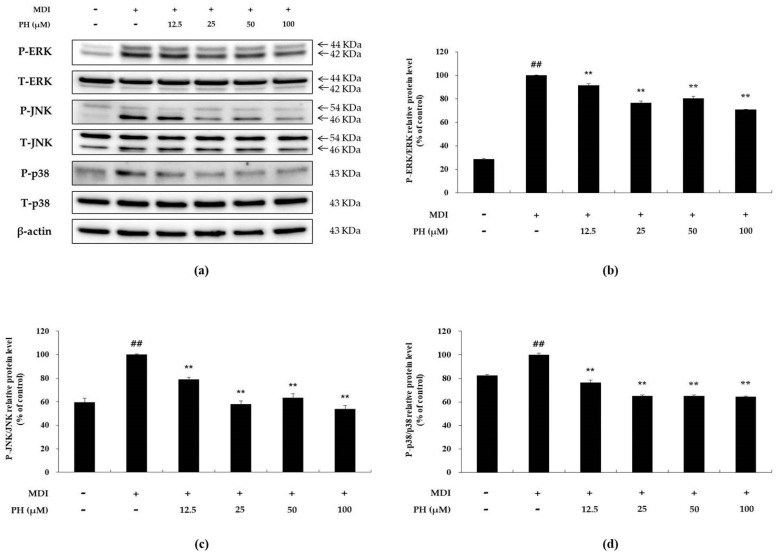
Effect of PH on the phosphorylation of MAPK. Adipocyte differentiation was induced in post-confluent cells using MDI differentiation medium, and the cells were treated with indicated concentrations of PH for 30 min. (**a**) The Western blot result of P-ERK/ERK, P-JNK/JNK, P-p38/p38, and β-actin in 3T3-L1 adipocytes. The relative protein levels of (**b**) P-ERK/ERK, (**c**) P-JNK/JNK, and (**d**) P-p38/p38 are presented as the mean ± standard deviation of three independent measurements using the ImageJ program. The results of the relative protein levels are expressed as percentages relative to the control cells treated with MDI alone. ** *p* < 0.01 indicates significant differences versus the MDI-only control. ## *p* < 0.01 indicates significant differences versus an untreated control.

Western blot analysis was performed to determine whether PH inhibits the phosphorylation of ERK (P-ERK), JNK (P-JNK), and p38 (P-p38) in differentiation-induced 3T3-L1 cells. P-ERK, P-JNK, and P-p38 were upregulated in the differentiation-induced cell control compared with their expression in the untreated control. In contrast, these levels were reduced in the PH-treated group compared with those in the differentiation-induced cell control group (Figure 4). Thus, the results indicate that PH attenuates adipogenesis via the inhibition of MAPK phosphorylation in the MAPK signaling pathway.

### 2.5. Effect of PH on the AKT-Related Signaling Pathways

**Figure 5:**
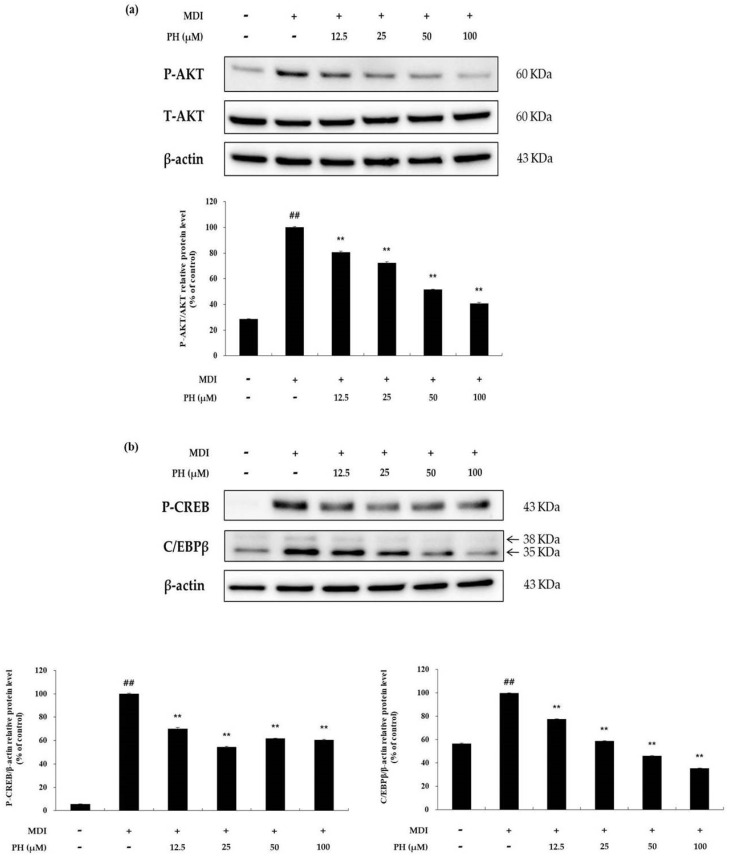
Effect of PH on the AKT-related signaling pathways. Adipocyte differentiation was induced in post-confluent cells using MDI differentiation medium, and the cells were treated with indicated concentrations of PH for 30 min (AKT) and 2 h (P-CREB, C/EBPβ). (**a**) Results of Western blotting and protein levels of P-AKT/AKT and β-actin. (**b**) Results of Western blotting and protein levels of P-CREB, C/EBPβ, and β-actin in 3T3-L1 adipocytes. The relative protein levels of P-AKT/AKT, P-CREB/β-actin, and C/EBPβ/β-actin are presented as the mean ± standard deviation of three independent measurements using the ImageJ program. The results in the relative protein levels are expressed as percentages relative to the control cells treated with MDI alone. ** *p* < 0.01 indicates significant differences versus the MDI-only control. ## *p* < 0.01 indicates significant differences versus an untreated control.

To confirm the effect of PH on the phosphorylation of AKT, the level of P-AKT protein was determined using Western blot analysis. As shown in Figure 5a, P-AKT was markedly higher in the differentiation-induced cell control than in the untreated control, whereas it was significantly decreased by PH treatment in a concentration-dependent manner. P-AKT affects the expression of CREB phosphorylation (P-CREB) and C/EBPβ, which are adipogenesis-related factors. The results showed that P-CREB and C/EBPβ protein expression was reduced by PH treatment compared with that in the differentiation-induced cell control (Figure 5b).

### 2.6. Effect of PH on the AMPK Signaling Pathway

**Figure 6:**
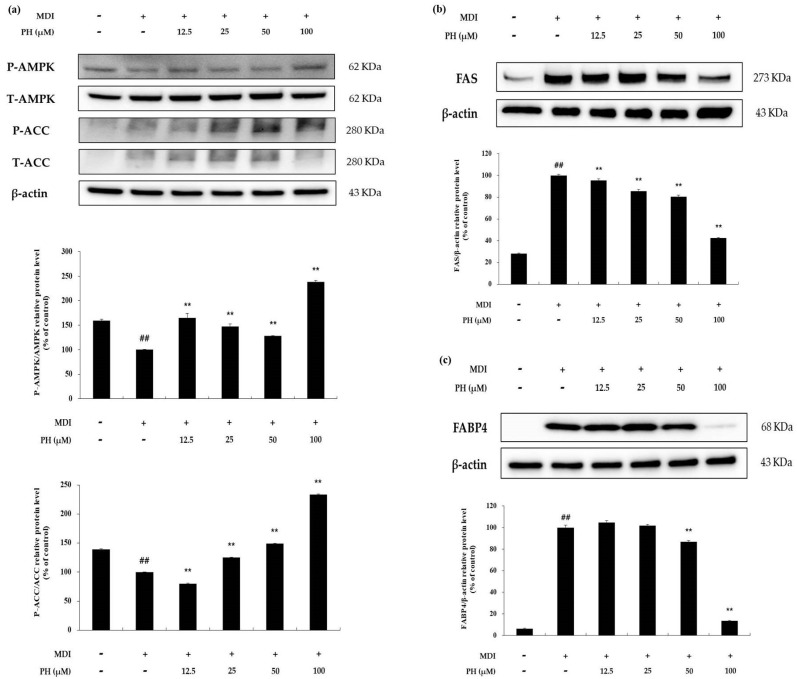
Effect of PH on the AMPK signaling pathway. Adipocyte differentiation was induced in post-confluent cells using MDI differentiation medium, followed by treatment with the indicated concentrations of PH for 6 days (AMPK, ACC) and 8 days (FAS, FABP4). (**a**) Results of the Western blotting analysis and the protein levels of P-AMPK/AMPK, P-ACC/ACC, and β-actin. (**b**) Results of the Western blotting analysis and the protein levels of FAS and β-actin in 3T3-L1 adipocytes. (**c**) Results of the Western blotting analysis and the protein levels of FABP4 and β-actin in 3T3-L1 adipocytes. The relative protein levels of P-AMPK/AMPK, P-ACC/ACC, FAS/β-actin, and FABP4/β-actin are presented as the mean ± standard deviation of three independent measurements using the ImageJ program. The relative protein levels are expressed as percentages relative to the protein levels of control cells treated with MDI alone. ** *p* < 0.01 indicates significant differences versus the MDI-only control. ## *p* < 0.01 indicates significant differences versus an untreated control.

To determine the effect of PH on AMP-activated protein kinase (AMPK) activation, the phosphorylation of AMPK (P-AMPK) and ACC (P-ACC) was detected using Western blot analysis. As shown in Figure 6a, P-AMPK and P-ACC were increased by PH treatment compared with the differentiation-induced cell control. In particular, P-AMPK and P-ACC protein levels were increased by 2.4-fold and 2.3-fold, respectively, after treatment with 100 μM PH. To evaluate the effect of these increases, 3T3-L1 cells were differentiated for 8 days and the expression of lipogenesis-related proteins was determined. The protein expression of FAS and FABP4 was reduced by PH treatment (Figure 6b). These results suggest that PH inhibits lipogenesis in differentiated 3T3-L1 cells by activating the AMPK signaling pathway.

## 3. Discussion

Adipose tissue plays an important role in maintaining homeostasis in the body through the secretion of various endocrine hormones and cytokines, as well as via energy storage and metabolism [4,5]. Therefore, an increase in adipocyte size (hypertrophy) and their excessive production (hyperplasia) as a result of obesity affect body homeostasis and eventually lead to complications, such as type 2 diabetes, hypertension, hyperlipidemia, cardiovascular disease, and cancer [6,7,8]. Therefore, identifying substances that modulate adipogenesis and studying their mechanisms of action is a possible therapeutic strategy in the treatment of obesity.

Stilbenoid is a polyphenol compound that naturally occurs in various plant species, possesses the basic skeleton of the stilbene structure (C6–C2–C6), and exists in various forms as a derivative of resveratrol [40]. Its bioactive properties include anti-inflammatory, antioxidative, neuroprotective, cardioprotective, anticancer, and anti-obesity effects [34,35,36,37,38,39]. However, the bioavailability and bioactivity of resveratrol are reduced owing to its instability in the environment (sensitivity to UVR, oxygen, alkaline pH, and high temperatures). Therefore, many resveratrol derivatives, especially methylated compounds, have been widely studied [41,42,43,44].

This mechanistic study focused on the anti-adipogenic effects of pinostilbene. The signaling pathways blocked by PH in 3T3-L1 adipocytes were also elucidated. The results showed that PH effectively attenuated adipogenesis in 3T3-L1 adipocytes. To determine the effects of PH on adipogenesis, lipid accumulation and TG content were analyzed using ORO staining and a TG quantification assay kit. In addition, Western blotting was performed to confirm the expression of adipogenesis-related transcription factors, and the signaling pathways involved were elucidated.

LDH is a stable enzyme that exists in the cytoplasm and cannot normally pass through the cell membrane. It is released into the medium when the cell membrane is damaged or during cell death [63]. Therefore, LDH release assays were performed to confirm the non-cytotoxic concentrations of PH; the results showed no toxicity at 12.5, 25, 50, and 100 μM concentrations of PH (Figure 1c). Mature adipocytes form lipid droplets due to the accumulation of lipids, including triglycerides [64]. As shown in Figure 2, treatment with PH led to the inhibition of lipid accumulation and TG content on day 8. These results suggest that PH is associated with the inhibition of adipogenesis through the inhibition of lipid accumulation and TG production at non-cytotoxic concentrations.

According to recent studies, adipogenesis is regulated by key transcription factors, such as C/EBPα, PPARγ, SREBP-1c, FAS, and FABP4 [17,18]. Our analysis of the effects of PH revealed that the protein expression of these transcription factors decreased with increasing concentrations of PH (Figure 3).

A mechanism study was performed to determine whether the activation of any of the several adipogenesis-related mechanisms is inhibited by PH, and, if so, which one. The MAPK signaling pathway is activated by various extracellular stimuli to induce various intracellular responses through phosphorylation of specific sites, and its components include ERK, JNK, and p38. Recent studies have reported that the activation of the MAPK family affects preadipocyte differentiation in the early stages of adipogenesis [28,29]. The MAPK family member ERK plays an important role in cell proliferation and differentiation during adipogenesis. Activated ERK induces the differentiation of preadipocytes into adipocytes by inducing an increase in the expression of the adipogenic transcription factors C/EBPα, C/EBPβ, and PPARγ. [22,28,29]. In addition, p38 MAPK promotes cell differentiation in the early stages of adipogenesis [65,66]. Moreover, JNK MAPK is suggested to be both a negative and positive regulator of adipocyte differentiation [67]. In this study, we showed that phosphorylation of ERK, JNK, and p38 MAPK was decreased by PH treatment (Figure 4). Previous studies have reported that the AKT-dependent signaling pathway is associated with adipogenesis. CREB phosphorylation by P-AKT induces the activation of C/EBPβ in the nucleus, which then upregulates C/EBPα and PPARγ expression, resulting in adipogenesis [68]. In our study, PH significantly decreased the phosphorylation of AKT and showed a tendency to suppress P-CREB and C/EBPβ protein expression (Figure 5). Based on these results, it is suggested that PH suppresses downstream protein activity by inhibiting the phosphorylation of MAPK and AKT, and downregulates the expression of adipogenesis-related transcription factors, ultimately suppressing adipogenesis.

AMP-activated protein kinase (AMPK) regulates energy metabolism through the synthesis and degradation of fatty acids in the body and maintains energy homeostasis. AMPK is activated by phosphorylation at Thr172. Phosphorylated AMPK leads to acetyl-CoA carboxylase (ACC) inactivation, resulting in the attenuation of lipogenesis via the suppression of SREBP-1c, FABP4, and FAS protein expression during adipogenesis [69,70,71,72]. As a result, AMPK and ACC phosphorylation increased upon PH treatment. In addition, the phosphorylation of AMPK and ACC was markedly increased upon treatment with the highest concentration of PH (100 µM, Figure 6a). Moreover, we examined whether the AMPK pathway affects the expression of FAS and FABP4, which are involved in lipogenesis in the late stage of adipogenesis. The results showed that lipogenesis-related protein expression was decreased by PH treatment, suggesting that PH inhibits lipogenesis in mature adipocytes via the AMPK signaling pathway.

Recently, metabolic and pharmacokinetic studies to assess bioavailability have been conducted to confirm not only the biological activities of a specific substance but also its applicability in vivo and, possibly, in humans [73]. These studies may show different results depending on the species, administration route, dose, and pattern [73,74]. It has been reported that the pharmacokinetic profiles of resveratrol and pinostilbene are unfavorable [75,76]. The bioavailability of resveratrol was confirmed through well-established studies on metabolism and pharmacokinetics in rodents (mice and rats), dogs, and humans [43,73]. However, the bioavailability of pinostilbene was suggested through the study of pharmacokinetics in Sprague–Dawley rats [75]. Although pinostilbene is unfavorable in pharmacokinetic studies, metabolic studies have reported that it is a major metabolite of intestinal microbiota pterostilbene and is more stable than resveratrol [56,57]. Therefore, various mammalian and human experiments are required to obtain accurate results on the bioavailability of pinostilbene.

Recent studies have reported that pinostilbene and pterostilbene have similar bioactivities, including anticancer [50,51,58], antioxidant [45,61], and neuroprotective activities [44,52,53,54,55]. The in vivo and in vitro anti-obesity effects of pterostilbene have been previously studied [47,48,49]. However, this study is the first to evaluate the inhibitory effect of pinostilbene on adipogenesis in 3T3-L1 cells. These comparative studies of pterostilbene and its metabolite pinostilbene will play an important role in understanding the pharmacological effects of pterostilbene in in vivo metabolism.

In summary, we showed that PH inhibits lipid and triglyceride accumulation in mature adipocytes. Our mechanistic studies supported a model wherein PH downregulates adipogenesis and lipogenesis by regulating the expression of adipogenesis-related factors via the MAPK and AKT-dependent insulin signaling, and the AMPK signaling pathway. These findings suggest that PH may be used in the prevention of obesity and as a potential therapeutic agent for obesity-related metabolic disorders. However, for direct in vivo application, more studies on bioavailability are needed, and whether PH directly affects adipose tissue through absorption in the intestine should be confirmed.

## 4. Materials and Methods

### 4.1. Chemicals and Reagents

PH, dimethyl sulfoxide (DMSO), and the protease inhibitor cocktail were obtained from Sigma-Aldrich (St. Louis, MO, USA). Dulbecco’s Modified Eagle’s Medium (DMEM), fetal bovine serum (FBS), penicillin/streptomycin (P/S), and trypsin–ethylenediaminetetraacetic acid (T/E) were purchased from Thermo Fisher Scientific (Waltham, MA, USA). Bovine calf serum (B/S) was obtained from Gibco™ (Grand Island, MD, USA). Tris-buffered saline (TBS), phosphate-buffered saline (PBS), enhanced chemiluminescence (ECL) kits, and radioimmunoprecipitation assay (RIPA) buffer were obtained from Biosesang (Seongnam, Gyeonggi-do, Korea). Lactate dehydrogenase (LDH), the cytotoxicity assay kit, and the triglyceride quantification assay kit were purchased from DoGenBio (Guro-gu, Seoul, Korea). The 2X Laemmli sample buffer was obtained from Bio-Rad (Hercules, CA, USA). Primary antibodies against FABP4, FAS, C/EBPα, C/EBPβ, PPARγ, phosphor-Ser133-CREB, phospho-Thr172-AMPK, total-AMPK, phosphor-Ser79-ACC, total-ACC, phosphor-Thr180/Tyr182-p38, total-p38, phospho-Thr183/Tyr185-SAPK/JNK, total-JNK, phospho-Thr202/Tyr204-p44/42/ERK, total-ERK, phospho-Ser473-AKT, total-AKT, and β-actin were obtained from Cell Signaling Technology (Danvers, MA, USA). Antibodies against sterol regulatory element-binding protein-1c (SREBP-1c) were obtained from BD Biosciences (San Jose, CA, USA). Protein levels were quantified and graphed using the ImageJ software (NIH, Bethesda, MD, USA). All reagents used were of analytical grade.

### 4.2. Cell Culture

3T3-L1 preadipocytes were obtained from the American Type Culture Collection (Rockville, MD, USA). The cells were maintained in DMEM containing 10% bovine calf serum and 1% penicillin/streptomycin at 37 °C under 95% air and 5% CO_2_. The culture medium was replaced every alternate day until 70–80% confluence was reached, and cells were sub-cultured every 3–4 days.

### 4.3. Adipocyte Differentiation

3T3-L1 preadipocytes were seeded into 24-well plates at a density of 5 × 10^4^ cells/well in B/S medium (DMEM, 10% B/S, 1% P/S) and maintained for 2 days after reaching 100% confluency. After 2 days of confluence (day 0), 3T3-L1 preadipocyte differentiation was induced by MDI differentiation medium containing DMEM, 1% P/S, 10% FBS, 0.5 mM 3-isobutyl-1-methylxanthine (IBMX), 1 μM dexamethasone (DEX), and 10 μg/mL insulin for an additional 2 days. On day 2, cells were incubated in fresh differentiation medium containing DMEM, 1% P/S, 10% FBS, and 10 μg/mL insulin for another 2 days. The cells were then maintained in post-differentiation medium (DMEM, 10% FBS, 1% P/S), which was replaced with fresh medium every 2 days until day 8. PH (12.5, 25, 50, and 100 μM) was added to the cells along with differentiation medium until differentiation was complete.

### 4.4. Measurement of Cell Cytotoxicity

3T3-L1 preadipocytes were seeded in 24-well plates and incubated until post-confluence. Subsequently, the cells were treated with PH (12.5, 25, 50, 100, and 200 μM) along with MDI differentiation medium (DMEM, 1% P/S, 10% FBS, 0.5 mM IBMX, 1 μM, and 10 μg/mL insulin) for 48 h. After incubation, the culture supernatant and LDH activity in the extracellular medium were quantified using the EZ-LDH assay kit to evaluate cytotoxicity in accordance with the manufacturer’s instructions. The culture medium was used as a volume control (blank), and the absorbance of each well was determined at 450 nm using a spectrophotometer.

### 4.5. Oil Red O Staining and Triglyceride Analysis

When lipid droplets are formed through adipocyte differentiation, the amount of lipid accumulation can be measured by the ORO staining assay. The cells in 24-well plates were gently washed twice with PBS and fixed with 10% formalin for at least 30 min. Subsequently, the fixed cells were washed gently with deionized water and treated with the prepared ORO working solution, which was added to each well. The cells were then stained for 30 min at 25 °C. The employed ORO working solution was obtained by diluting a twice-filtered ORO stock solution (2-propanol containing 0.5% Oil Red O) with deionized water in a 6:4 ratio. After staining, the staining solution was removed, washed with deionized water, and incubated in PBS. Images of the stained lipid droplets were observed and photographed under a microscope. To quantify the lipid accumulation, 2-propanol (300 μL) was added to each well and incubated in a shaker for 30 min. The absorbance of each well was measured at 540 nm using a spectrophotometer. TG content was measured and quantified using the EZ-TG quantification assay kit (DoGenBio, Guro-gu, Seoul, Korea) in accordance with the manufacturer’s instructions. The lipid and triglyceride contents were analyzed using adipocytes, the differentiation of which was induced from day 0 to 8.

### 4.6. Western Blot Analysis

3T3-L1 preadipocytes were seeded in 6-well plates and incubated until post-confluence. Thereafter, the cells were treated with PH (12.5, 25, 50, and 100 μM) and MDI differentiation medium (DMEM, 1% P/S, 10% FBS, 0.5 mM IBMX, 1 μM, and 10 μg/mL insulin), and then differentiated for different times to check the expression of various target proteins. The cells in each well were washed with cold PBS and lysed in 200 μL of RIPA buffer containing 1% protease inhibitor cocktail, 150 mM sodium chloride, 1% Triton X-100, 1% sodium deoxycholate, 0.1% SDS, 50 mM Tris-HCl, pH 7.5, and 2 mM EDTA. The cell extract solution was vortexed and centrifuged at 11,000× *g* to collect the supernatant. The protein level of the supernatant was quantified with a BCA kit, and an equal amount of protein (15–30 μg) was used to prepare each Western loading sample. The Western blot samples were heated for 5 min and cooled on ice. Equal amounts of proteins in each Western blot sample were separated using 6–12% sodium dodecyl sulfate-polyacrylamide gels and transferred to a polyvinylidene difluoride membrane using a Trans-blot Turbo (Bio-Rad, Hercules, CA, USA). The protein-transferred membrane was blocked for 1 h at 25 °C using 5% skim milk in TBST (20 mM Tris base, 137 mM NaCl, pH 7.6, and 0.1% Tween-20), washed six times with TBST each time for 10 min, and subsequently incubated overnight at 4 °C with primary antibodies (1:1000). The membrane was then washed six times with TBST and incubated with secondary antibodies (1:1000) at 25 °C for 1 h, followed by six washes with TBST. The secondary antibody binding the target protein was detected using an ECL kit.

### 4.7. Statistical Analysis

All experiments were carried out in triplicate, and the results are expressed as the mean ± SD. Differences between the control and treatment groups were evaluated by one-way analysis of variance (ANOVA) using GraphPad Prism. The significance value was determined: * *p* < 0.05, ** *p* < 0.01 versus MDI alone, ## *p* < 0.01 versus untreated cell control.

## Data Availability

The data presented in this study are available on request from the corresponding author.
